# Supplementary material for: Comparison of collection methods for Phlebotomus argentipes sand flies to use in a molecular xenomonitoring system for the surveillance of visceral leishmaniasis
Source: PLoS Negl Trop Dis. 2023 Sep 1;17(9):e0011200. doi: 10.1371/journal.pntd.0011200 (PMC10501600; doi:10.1371/journal.pntd.0011200)
Supplement: S1 Table — (DOCX) [file pntd.0011200.s001.docx]

| Rounds1-4 | Collection Method | | | **Total** |
| --- | --- | --- | --- | --- |
|  | CDC | MVA | PKP |  |
| **Total sand fly females** | 1621 (48.5) | 810 (24.3) | 908 (27.0) | **3,339** |
| *P. argentipes* females | 866 (44.8) | 501 (25.9) | 567 (29.3) | **1,934** |
| *P. papatasi* females | 24 (23.3) | 47 (45.6) | 32 (31.1) | **103** |
| *Sergentomyia babu* females | 704 (58.9) | 224 (18.7) | 267 (22.3) | **1195** |
| Other/Unknown females | 27 (25.2) | 38 (35.5) | 42 (39.3) | **107** |
| **Total sand fly males** | 2293 (66.1) | 558 (16.1) | 619 (17.8) | **3,470** |
| **Total sand flies** | 3914 (57.5) | 1368 (20.1) | 1527 (22.4) | **6,809** |
| **Total female mosquitoes** | 3085 (37.7) | 3193 (39.1) | 1896 (23.2) | **8174** |
