# Supplementary material for: Comparison of collection methods for Phlebotomus argentipes sand flies to use in a molecular xenomonitoring system for the surveillance of visceral leishmaniasis
Source: PLoS Negl Trop Dis. 2023 Sep 1;17(9):e0011200. doi: 10.1371/journal.pntd.0011200 (PMC10501600; doi:10.1371/journal.pntd.0011200)
Supplement: S2 Table — (DOCX) [file pntd.0011200.s002.docx]

| Collection Round | *P. argentipes* | *S. babu* | Female Sand Flies | Female Mosquitoes | Total Sand Flies |
| --- | --- | --- | --- | --- | --- |
| **Round 1**  (June 25 – July 12) | **294** | **293** | **641** | **969** | **1207** |
| *Bishambharpur* | 144 | 76 | 230 | 198 | 459 |
| *Dharampur* | 77 | 104 | 196 | 186 | 348 |
| *Rampur Jagdish* | 41 | 69 | 131 | 451 | 255 |
| *Ruchanpura* | 32 | 44 | 84 | 134 | 145 |
| **Round 2**  (July 16 – Aug 2) | **633** | **516** | **1220** | **1825** | **2528** |
| *Bishambharpur* | 95 | 116 | 229 | 120 | 580 |
| *Dharampur* | 200 | 154 | 372 | 657 | 743 |
| *Rampur Jagdish* | 167 | 85 | 279 | 641 | 529 |
| *Ruchanpura* | 171 | 161 | 340 | 407 | 676 |
| **Round 3**  (Aug 6 – Aug 24) | **693** | **263** | **1010** | **2586** | **1961** |
| *Bishambharpur* | 229 | 26 | 257 | 247 | 644 |
| *Dharampur* | 188 | 87 | 280 | 1099 | 462 |
| *Rampur Jagdish* | 162 | 36 | 243 | 391 | 442 |
| *Ruchanpura* | 114 | 114 | 230 | 849 | 413 |
| **Total** | **1620** | **1072** | **2871** | **5380** | **5696** |
