# Supplementary material for: Comparison of collection methods for Phlebotomus argentipes sand flies to use in a molecular xenomonitoring system for the surveillance of visceral leishmaniasis
Source: PLoS Negl Trop Dis. 2023 Sep 1;17(9):e0011200. doi: 10.1371/journal.pntd.0011200 (PMC10501600; doi:10.1371/journal.pntd.0011200)
Supplement: S3 Table — (DOCX) [file pntd.0011200.s003.docx]

| Rounds 1-3 | Collection Method | | | **Total** |
| --- | --- | --- | --- | --- |
|  | CDC-LT | MVA | PKP |  |
| **Total sand fly females** | 1441 (50.2) | 685 (23.9) | 745 (25.9) | **2871** |
| *P. argentipes* females | 726 (44.8) | 426 (26.3) | 468 (28.9) | **1620** |
| *P. papatasi* females | 24 (28.2) | 34 (40.0) | 85 (31.8) | **85** |
| *Sergentomyia babu* females | 664 (61.9) | 187 (17.4) | 221 (20.6) | **1072** |
| Other/Unknown females | 27 (28.7) | 38 (40.4) | 29 (30.9) | **94** |
| **Total sand fly males** | 2019 (71.5) | 371 (13.1) | 435 (15.4) | **2825** |
| **Total sand flies** | 3460 (60.7) | 1056 (18.5) | 1180 (20.7) | **5696** |
| **Total female mosquitoes** | 1843 (34.3) | 2097 (39.0) | 1140 (26.8) | **5380** |
