# Supplementary figures and images for: Comparison of collection methods for Phlebotomus argentipes sand flies to use in a molecular xenomonitoring system for the surveillance of visceral leishmaniasis
Source: PLoS Negl Trop Dis. 2023 Sep 1;17(9):e0011200. doi: 10.1371/journal.pntd.0011200 (PMC10501600; doi:10.1371/journal.pntd.0011200)

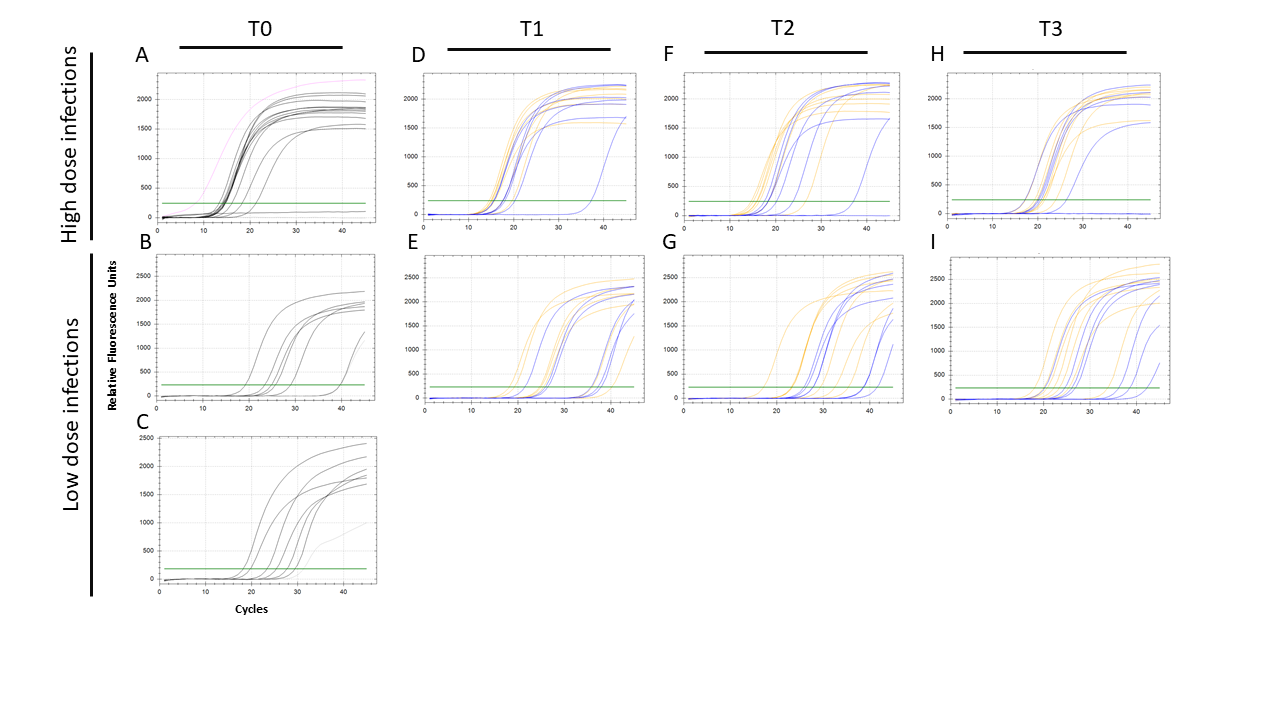

Supplement: S1 Fig — Flies were killed and stored under simulated conditions for Prokopack aspirator (PKP: orange) or CDC-LT collection (CDC: blue) and kept in a cool bag for up to 7 hours (Table 1). (A, B, C) T0 amplification curves for infected sand flies (black), processed immediately after killing (A: High dose infection; B & C: Low dose infections), including a positive control of 1x10^6^ cultured Log phase L. donovani promastigotes (magenta) and a non-template negative control of DNA-free water (grey). (D, E) T1 amplification curves for high (D) and low (E) infected sand flies processed after exposure of killed flies to 40°C for 30 min (PKP) or 16 hours (CDC). (F, G) T2 amplification curves for high (F) and low (G) infected sand flies processed after exposure of killed flies to 40°C for 30 min and 4°C for 3 hours (PKP) or 40°C for 16 hours and 4°C for 3 hours (CDC). (H, I) T3 amplification curves for high (H) and low (I) infected sand flies killed and stored under ‘worst case scenario’ field conditions of 40°C for 30 min followed by 7 hours at 4°C (PKP), or 40°C for 16 hours followed by 7 hours at 4°C (CDC) before processing. (TIF) [file pntd.0011200.s004.tif]
